# No Difference in Penetrance between Truncating and Missense/Aberrant Splicing Pathogenic Variants in *MLH1* and *MSH2*: A Prospective Lynch Syndrome Database Study

**DOI:** 10.3390/jcm10132856

**Published:** 2021-06-28

**Authors:** Mev Dominguez-Valentin, John-Paul Plazzer, Julian R. Sampson, Christoph Engel, Stefan Aretz, Mark A. Jenkins, Lone Sunde, Inge Bernstein, Gabriel Capella, Francesc Balaguer, Finlay Macrae, Ingrid M. Winship, Huw Thomas, Dafydd Gareth Evans, John Burn, Marc Greenblatt, Wouter H. de Vos tot Nederveen Cappel, Rolf H. Sijmons, Maartje Nielsen, Lucio Bertario, Bernardo Bonanni, Maria Grazia Tibiletti, Giulia Martina Cavestro, Annika Lindblom, Adriana Della Valle, Francisco Lopez-Kostner, Karin Alvarez, Nathan Gluck, Lior Katz, Karl Heinimann, Carlos A. Vaccaro, Sigve Nakken, Eivind Hovig, Kate Green, Fiona Lalloo, James Hill, Hans F. A. Vasen, Claudia Perne, Reinhard Büttner, Heike Görgens, Elke Holinski-Feder, Monika Morak, Stefanie Holzapfel, Robert Hüneburg, Magnus von Knebel Doeberitz, Markus Loeffler, Nils Rahner, Jürgen Weitz, Verena Steinke-Lange, Wolff Schmiegel, Deepak Vangala, Emma J. Crosbie, Marta Pineda, Matilde Navarro, Joan Brunet, Leticia Moreira, Ariadna Sánchez, Miquel Serra-Burriel, Miriam Mints, Revital Kariv, Guy Rosner, Tamara Alejandra Piñero, Walter Hernán Pavicic, Pablo Kalfayan, Sanne W. ten Broeke, Jukka-Pekka Mecklin, Kirsi Pylvänäinen, Laura Renkonen-Sinisalo, Anna Lepistö, Päivi Peltomäki, John L. Hopper, Aung Ko Win, Daniel D. Buchanan, Noralane M. Lindor, Steven Gallinger, Loïc Le Marchand, Polly A. Newcomb, Jane C. Figueiredo, Stephen N. Thibodeau, Christina Therkildsen, Thomas V. O. Hansen, Lars Lindberg, Einar Andreas Rødland, Florencia Neffa, Patricia Esperon, Douglas Tjandra, Gabriela Möslein, Toni T. Seppälä, Pål Møller

**Affiliations:** 1 Department of Tumor Biology, Institute of Cancer Research, The Norwegian Radium Hospital, 0379 Oslo, Norway; sigven@ifi.uio.no (S.N.); ehovig@ifi.uio.no (E.H.); einarro@ifi.uio.no (E.A.R.); moller.pal@gmail.com (P.M.); 2 European Hereditary Tumour Group (EHTG), c/o Lindsays, Caledonian Exchange 19A Canning Street, Edinburgh EH3 8HE, UK; Sampson@cardiff.ac.uk (J.R.S.); christoph.engel@imise.uni-leipzig.de (C.E.); capella.gabriel@gmail.com (G.C.); john.burn@newcastle.ac.uk (J.B.); r.h.sijmons@medgen.umcg.nl (R.H.S.); jukka-pekka.mecklin@ksshp.fi (J.-P.M.); gabriela.moeslein@helios-kliniken.de (G.M.); toni.seppala@fimnet.fi (T.T.S.); 3 The International Society for Gastrointestinal Hereditary Tumours (InSiGHT), The Polyposis Registry, St Mark’s Hospital, Watford Road, Harrow, Middlesex HA1 3UJ, UK; johnpaul.plazzer@gmail.com (J.-P.P.); finlay.macrae@mh.org.au (F.M.); holinski-feder@mgz-muenchen.de (E.H.-F.); 4 Department of Medicine, Colorectal Medicine and Genetics, The Royal Melbourne Hospital, Melbourne, VIC 3050, Australia; dptjandra@gmail.com; 5 Institute of Medical Genetics, Division of Cancer and Genetics, Cardiff University School of Medicine, Heath Park, Cardiff CF14 4XN, UK; 6 Institute for Medical Informatics, Statistics and Epidemiology, University of Leipzig, 04107 Leipzig, Germany; markus.loeffler@imise.uni-leipzig.de; 7 Institute of Human Genetics, National Center for Hereditary Tumor Syndromes, Medical Faculty, University Hospital Bonn, University of Bonn, 53127 Bonn, Germany; stefan.aretz@uni-bonn.de (S.A.); claudia.perne@ukbonn.de (C.P.); stefanie.holzapfel@ukb.uni-bonn.de (S.H.); 8 Melbourne School of Population and Global Health, Centre for Epidemiology and Biostatistics, The University of Melbourne, Parkville, VIC 3010, Australia; m.jenkins@unimelb.edu.au (M.A.J.); j.hopper@unimelb.edu.au (J.L.H.); awin@unimelb.edu.au (A.K.W.); 9 Department of Clinical Genetics, Aalborg University Hospital, 9000 Aalborg, Denmark; l.sunde@rn.dk; 10 Department of Biomedicine, Aarhus University, DK-8000 Aarhus, Denmark; 11 Department of Surgical Gastroenterology, Aalborg University Hospital, Aalborg University, 9100 Aalborg, Denmark; i.bernstein@rn.dk; 12 Department of Clinical Medicine, Aalborg University Hospital, Aalborg University, 9100 Aalborg, Denmark; 13 Hereditary Cancer Program, Institut Català d’Oncologia-IDIBELL, L, Hospitalet de Llobregat, 08908 Barcelona, Spain; mpineda@iconcologia.net (M.P.); mnavarrogarcia@iconcologia.net (M.N.); jbrunet@iconcologia.net (J.B.); 14 Gastroenterology Department, Hospital Clínic de Barcelona, Centro de Investigación Biomédica en Red de Enfermedades Hepáticas y Digestivas (CIBERehd), Institut d’Investigacions Biomediques August Pi i Sunyer (IDIBAPS), Universitat de Barcelona, 08036 Barcelona, Spain; fprunes@clinic.cat (F.B.); lmoreira@clinic.cat (L.M.); asanchezg@clinic.cat (A.S.); 15 Department of Genomic Medicine, Royal Melbourne Hospital, University of Melbourne, Melbourne, VIC 3052, Australia; ingrid.winship@mh.org.au; 16 Department of Medicine, Royal Melbourne Hospital, University of Melbourne, Melbourne, VIC 3052, Australia; 17 Department of Surgery and Cancer, St Mark’s Hospital, Imperial College London, London HA1 3UJ, UK; huw.thomas@imperial.ac.uk; 18 Manchester Centre for Genomic Medicine, Manchester University NHS Foundation Trust, Manchester M13 9WL, UK; Gareth.Evans@mft.nhs.uk (D.G.E.); Kate.Green@mft.nhs.uk (K.G.); Fiona.Lalloo@mft.nhs.uk (F.L.); 19 Faculty of Medical Sciences, Newcastle University, Newcastle upon Tyne NE1 7RU, UK; 20 Larner College of Medicine, University of Vermont, Burlington, VT 05405, USA; Marc.Greenblatt@uvmhealth.org; 21 Department of Gastroenterology and Hepatology, Isala Clinics, 8015 Zwolle, The Netherlands; w.h.de.vos@isala.nl; 22 Department of Genetics, University Medical Center Groningen, University of Groningen, 9713GZ Groningen, The Netherlands; 23 Department of Clinical Genetics, Leids Universitair Medisch Centrum, 2300RC Leiden, The Netherlands; M.Nielsen@lumc.nl (M.N.); s.w.ten.broeke@umcg.nl (S.W.t.B.); 24 Division of Cancer Prevention and Genetics, IEO, European Institute of Oncology, Fondazione IRCCS Istituto Nazionale dei Tumori, IRCCS, 20141 Milan, Italy; lucio.bertario@gmail.com; 25 Division of Cancer Prevention and Genetics, IEO, European Institute of Oncology, IRCCS, 20141 Milan, Italy; bernardo.bonanni@ieo.it; 26 Ospedale di Circolo ASST Settelaghi, Centro di Ricerca Tumori Eredo-Familiari, Università dell’Insubria, 21100 Varese, Italy; mariagrazia.tibiletti@asst-settelaghi.it; 27 Gastroenterology and Gastrointestinal Endoscopy Unit, San Raffaele Scientific Institute, Vita-Salute San Raffaele University, 20132 Milan, Italy; cavestro.giuliamartina@hsr.it; 28 Department of Molecular Medicine and Surgery, Karolinska Institutet, 171 76 Stockholm, Sweden; annika.lindblom@ki.se; 29 Grupo Colaborativo Uruguayo, Investigación de Afecciones Oncológicas Hereditarias (GCU), Hospital Fuerzas Armadas, Montevideo 11600, Uruguay; adrianadellav@gmail.com (A.D.V.); floneffa@gmail.com (F.N.); pesperon@fq.edu.uy (P.E.); 30 Programa Cáncer Heredo Familiar, Clínica Universidad de los Andes, Santiago 7550000, Chile; franciscolopezk@gmail.com (F.L.-K.); karinalvarezvalenzuela@gmail.com (K.A.); 31 Department of Gastroenterology, Sackler Faculty of Medicine, Tel-Aviv Sourasky Medical Center, Tel-Aviv University, Tel-Aviv 64259, Israel; nathang@tlvmc.gov.il (N.G.); revitalk@tlvmc.gov.il (R.K.); guyr@tlvmc.gov.il (G.R.); 32 The Department of Gastroenterology, Gastro-Oncology Unit, High Risk and GI Cancer Prevention Clinic, Sheba Medical Center, Sheba 91120, Israel; liorkatz5346@gmail.com; 33 Medical Genetics, Institute for Medical Genetics and Pathology, University Hospital Basel, 4031 Basel, Switzerland; Karl.Heinimann@usb.ch; 34 Hereditary Cancer Program (PROCANHE), Hospital Italiano de Buenos Aires, Buenos Aires C1199ABB, Argentina; carlos.vaccaro@hospitalitaliano.org.ar (C.A.V.); tamara.pinero@hospitalitaliano.org.ar (T.A.P.); walter.pavicic@gmail.com (W.H.P.); pablo.kalfayan@hospitalitaliano.org.ar (P.K.); 35 Instituto de Medicina Traslacional e Ingenieria Biomedica (IMTIB), CONICET IU, Hospital Italiano de Buenos Aires, Buenos Aires C1199ABB, Argentina; 36 Centre for Cancer Cell Reprogramming (CanCell), Institute of Clinical Medicine, Faculty of Medicine, University of Oslo, 4950 Oslo, Norway; 37 Department of Informatics, Centre for Bioinformatics, University of Oslo, 0316 Oslo, Norway; 38 Department of Surgery, Central Manchester University Hospitals NHS, Foundation Trust, University of Manchester, London M13 9WL, UK; james.hill@cmft.nhs.uk; 39 Department of Gastroenterology and Hepatology, Leiden University Medical Centre, 2333 Leiden, The Netherlands; hfavasen@stoet.nl; 40 Institute of Pathology, University of Cologne, 50937 Cologne, Germany; reinhard.buettner@uk-koeln.de; 41 Department of Surgery, Technische Universität Dresden, 01062 Dresden, Germany; Heike.Schackert28@gmail.com (H.G.); juergen.weitz@uniklinikum-dresden.de (J.W.); 42 Campus Innenstadt, Medizinische Klinik und Poliklinik IV, Klinikum der Universität München, 80336 Munich, Germany; monika.morak@gmx.de (M.M.); Verena.Steinke-Lange@mgz-muenchen.de (V.S.-L.); 43 Center of Medical Genetics, 80335 Munich, Germany; 44 Department of Internal Medicine, University Hospital Bonn, 53127 Bonn, Germany; robert.hueneburg@ukbonn.de; 45 Department of Applied Tumour Biology, Institute of Pathology, University Hospital Heidelberg, 69120 Heidelberg, Germany; magnus.knebel-doeberitz@med.uni-heidelberg.de; 46 Cooperation Unit Applied Tumour Biology, German Cancer Research Center (DKFZ), 69120 Heidelberg, Germany; 47 Medical School, Institute of Human Genetics, Heinrich-Heine-University, 40225 Dusseldorf, Germany; nils.rahner@uni-duesseldorf.de; 48 Department of Medicine, Knappschaftskrankenhaus, Ruhr-University Bochum, D-44789 Bochum, Germany; meduni-kkh@ruhr-uni-bochum.de (W.S.); deepak.vangala@rub.de (D.V.); 49 Gynaecological Oncology Research Group, Manchester University NHS Foundation Trust, Manchester, UK and Division of Cancer Sciences, University of Manchester, Manchester M20 4GJ, UK; Emma.Crosbie@manchester.ac.uk; 50 Centre de Recerca en Economia i Salut (CRES-UPF), Universitat de Barcelona, 08002 Barcelona, Spain; miquel.serra@barcelonagse.eu; 51 Division of Obstetrics and Gyneacology, Department of Women’s and Children’s Health, Karolinska Institutet, Karolinska University Hospital, Solna, 171 77 Stockholm, Sweden; miriam.mints@ki.se; 52 Departments of Surgery, Central Finland Hospital Nova, University of Jyväskylä, 40620 Jyväskylä, Finland; 53 Department of Education and Science, Sport and Health Sciences, Central Finland Hospital Nova, University of Jyväskylä, FI-40014 Jyväskylä, Finland; kirsi.pylvanainen@ksshp.fi; 54 Applied Tumour Genomics Research Program, University of Helsinki, 00014 Helsinki, Finland; laura.renkonen-sinisalo@hus.fi (L.R.-S.); anna.lepisto@hus.fi (A.L.); 55 Department of Gastrointestinal Surgery, Helsinki University Central Hospital, University of Helsinki, 00280 Helsinki, Finland; 56 Department of Medical and Clinical Genetics, University of Helsinki, 00014 Helsinki, Finland; peltomak@mappi.helsinki.fi; 57 Centre for Cancer Research, Faculty of Medicine, Dentistry and Health Sciences, University of Melbourne, Melbourne, VIC 3010, Australia; daniel.buchanan@unimelb.edu.au; 58 Colorectal Oncogenomics Group, Department of Clinical Pathology, The University of Melbourne, Parkville, VIC 3010, Australia; 59 Genomic Medicine and Family Cancer Clinic, Royal Melbourne Hospital, Parkville, VIC 3010, Australia; 60 Department of Health Science Research, Mayo Clinic Arizona, Phoenix, AZ 85054, USA; noralanelindor@gmail.com; 61 Lunenfeld-Tanenbaum Research Institute, Mount Sinai Hospital, University of Toronto, Toronto, ON M5G 1X5, Canada; sgallinger@rogers.com; 62 Cancer Center, University of Hawaii, Honolulu, HI 96813, USA; loic@cc.hawaii.edu; 63 Public Health Sciences Division, Fred Hutchinson Cancer Research Center, Seattle, WA 98109-1024, USA; pnewcomb@fredhutch.org; 64 Cedars-Sinai Medical Center, Los Angeles, CA 90048, USA; jane.figueiredo@cshs.org; 65 Department of Laboratory Medicine and Pathology, Mayo Clinic, Rochester, MN 55905, USA; sthibodeau@mayo.edu; 66 The Danish HNPCC Register, Clinical Research Centre, Copenhagen University Hospital, 2560 Hvidovre, Denmark; 2xtherkild@gmail.com; 67 Department of Clinical Genetics, Rigshospitalet, Copenhagen University Hospital, 2100 Copenhagen, Denmark; Thomas.Van.Overeem.Hansen@regionh.dk; 68 Gastro Unit, Copenhagen University Hospital, 2560 Hvidovre, Denmark; lars.joachim.lindberg@regionh.dk; 69 Surgical Center for Hereditary Tumors, Ev. Bethesda Khs Duisburg, University Witten-Herdecke, 58448 Herdecke, Germany; 70 Department of Surgical Oncology, Johns Hopkins Hospital, Baltimore, MA 21287, USA

**Keywords:** *MLH1*, *MSH2*, penetrance, cancer incidence, truncating, missense, aberrant splicing, Lynch syndrome

## Abstract

Background. Lynch syndrome is the most common genetic predisposition for hereditary cancer. Carriers of pathogenic changes in mismatch repair (MMR) genes have an increased risk of developing colorectal (CRC), endometrial, ovarian, urinary tract, prostate, and other cancers, depending on which gene is malfunctioning. In Lynch syndrome, differences in cancer incidence (penetrance) according to the gene involved have led to the stratification of cancer surveillance. By contrast, any differences in penetrance determined by the type of pathogenic variant remain unknown. Objective. To determine cumulative incidences of cancer in carriers of truncating and missense or aberrant splicing pathogenic variants of the *MLH1* and *MSH2* genes. Methods. Carriers of pathogenic variants of *MLH1* (*path_MLH1*) and *MSH2* (*path_MSH2*) genes filed in the Prospective Lynch Syndrome Database (PLSD) were categorized as truncating or missense/aberrant splicing according to the InSiGHT criteria for pathogenicity. Results. Among 5199 carriers, 1045 had missense or aberrant splicing variants, and 3930 had truncating variants. Prospective observation years for the two groups were 8205 and 34,141 years, respectively, after which there were no significant differences in incidences for cancer overall or for colorectal cancer or endometrial cancers separately. Conclusion. Truncating and missense or aberrant splicing pathogenic variants were associated with similar average cumulative incidences of cancer in carriers of *path MLH1* and *path_MSH2*.

## 1. Introduction

Lynch syndrome (LS) is a common, dominantly inherited cancer syndrome caused by pathogenic variants of mismatch repair genes (*path_MMR*) [1,2,3,4] and affects an estimated 1 in 300 individuals. *Path_MMR* carriers have increased incidences of cancers of the colon, rectum (often grouped as colorectal cancer, CRC), endometrium, ovaries, stomach, small bowel, bile duct, pancreas, and upper urinary tract [1,4,5,6]. The cancers may occur much earlier in life than their sporadic counterparts, and penetrance and expression vary by gene and by gender from very high to not measurable [7]. Factors considered likely to contribute to both incomplete penetrance and variation in cancer incidence in different organs include environmental factors, modifying genetic factors, and the nature of the pathogenic variants themselves. Genetic association studies have examined the relationship between variants elsewhere in the genome and cancer incidence in LS individuals and have suggested that SNPs at 8q23.3 (rs16892766) and 11q23.1 (rs3802842) are associated with increased LS CRC risk, especially for female *MLH1* carriers [8,9]. By contrast, a recent study did not find any risk-modifying effects of these SNPs in a cohort of 507 *PMS2* carriers [10]. Additional factors implicated in phenotypic variability in LS include epigenetic regulators, microRNAs, hormonal factors, acetyl-salicylic acid prophylaxis, smoking, and body mass index. In the current study, which addresses the question of whether penetrance varies according to the type of *path_MMR* variant, such modifying factors are not expected be stratified by the type of *path_MMR* variant.

According to the InSiGHT database (https://www.insight-group.org/variants/databases/, accessed on 12 February 2021), more than 3000 different pathogenic or likely pathogenic (class 5 or 4 and, therefore, clinically actionable) germline sequence variants have been deposited for the MMR genes, of which 40% have been identified in *MLH1*, 34% in *MSH2*, 18% in *MSH6*, and 8% in *PMS2* [11,12]. Approximately 50% of those in *MLH1* are missense variants [13,14,15,16], whereas most affecting *MSH2* are nonsense, frameshift, or splice site changes, which can be considered *a priori* to be pathogenic [13,14,15,16]. Pathogenic variants that result in aberrant splicing may be associated with lower penetrance compared to truncating variants of the same gene [17]. Recent studies have shown that some exonic missense variants (and some synonymous variants) cause disease through interference with the splicing machinery, adding complexity to the classification of variants [18,19,20]. The potential for clinically relevant associations with different types of germline variants in LS was illustrated by a recent study that reported a significantly better prognosis for CRC in LS patients who had missense or splice site *path_MMR* variants compared to those with frameshift or nonsense variants or large genomic rearrangements (overall survival 132.5 vs. 82.5 months) [21]. In contrast, one retrospective study suggested an increased risk for endometrial cancer in carriers of missense *path_MLH1* variants, but this was not seen in other cancers [6].

We here report prospectively observed, cumulative incidences of cancer in *path_MLH1* and *path_MSH2* carriers with truncating versus predicted missense and non-canonical aberrant splicing pathogenic variants to explore the hypothesis that carriers of truncating variants have higher cancer incidence.

## 2. Methods

### 2.1. The Prospective Lynch Syndrome Database (PLSD) Design

We analysed carriers of *path_MLH1* and *path_MSH2* variants from the PLSD. The PLSD design and its inclusion criteria have been described previously in detail [1,2,3,4,7]. In brief, the PLSD is an international prospective observational study including centres from 18 countries worldwide. Data were collected from the first prospectively planned and completed colonoscopy onwards, and all recruits had subsequent follow-up of one year or more. A detailed discussion of methods is given in Moller et al. and Seppälä et al. [7,22]. Time to first cancer after inclusion was calculated for each organ or group of organs. When calculating the time to any cancer (penetrance), only patients without any cancer prior to or at inclusion were counted. For each calculation, each patient was censored at the first event or last observation, whichever came first. The number of observation years and cancers in the 5-year groups were counted from 25 to 75 years and the corresponding annual cancer incidence rates by age group were calculated.

### 2.2. MMR Gene Variant Categorization

*Path_MLH1* and *path_MSH2* variants that were classified as clinically actionable (class 4 and 5) in the InSIGHT database [23] were grouped as: (1) truncating (including frameshift, nonsense, deletion of exon(s), and canonical splicing); (2) missense/aberrant splicing (aberrant splicing determined by splicing assay of intronic variants outside the canonical +/− 2(3) positions or exonic variants), and (3) others (including in-frame deletions or duplications, duplications of whole exons, initiation codon variants, intronic variants, and variants not compliant with any of the categories described). The groups of truncating and missense/aberrant splicing variants were used for calculations. As previously reported [1], the number of carriers with *path_MSH6* or *path_PMS2* variants were limited and considered insufficient for the analyses presented in this report.

### 2.3. Cancer Risk by Gene and Type of Genetic Variant

The cumulative incidence (Q) and the annual incidence rates (AIRs) by age were calculated as previously described [1]. In brief, Q was computed starting at age 25, assuming zero incidence rate before age 25, using the formula Q (age) = Q (age − 1) + (1 − Q (age − 1)) × AIR (age), where AIR (age) is the annual incidence rate as estimated from the corresponding 5-year interval. Confidence intervals were calculated as previously described [1].

### 2.4. Ethics Statement

All reporting centers exported de-identified data to the PLSD, and the patients had been followed up prospectively according to local clinical guidelines, as previously described [1,2,3,4,24,25].

## 3. Results

### 3.1. Characterization of Path_MLH1 and Path_MSH2 Genetic Variants

Numbers of carriers and follow-up times by gene, variant type—missense/aberrant splicing or truncating or other—are detailed in Table 1. In sum, 1045 carriers with missense/aberrant splicing variants were followed for an average of 7.9 years (95% CI (7.6–8.2)), 3930 carriers with truncating variants were followed for an average of 8.7 years (95% CI (8.5–8.9)), and 224 carriers had other types of variants (Table 1). Because carriers of *path_MLH1* and *path_MSH2* have different incidences of cancers [1], the incidences in this report were calculated for each gene separately.

For the *MLH1* gene, missense variants were more frequent (60.7%, 345/578) than aberrant splicing variants (40.3%, 233/578), while for the *MSH2* gene, aberrant splicing variants were more common than missense variants (75%, 350/467 vs. 26%, 117/467) (*p* > 0.05). Truncating variants affected both genes in an equal proportion (50% each). Within the set of truncating variants (*n* = 3930), the most common types with respect to variant consequence were exon or multi-exon deletions (32%, 1267/3930), followed by frameshift (27%, 1045/3930) and nonsense (24%, 932/3930). By the type of truncating variant and gene, exon or multi-exon deletions were the more frequent variant in *MLH1* (34.5%, 688/1995), followed by canonical splicing variants (25.1%, 501/1995), frameshift (24.1%, 482/1995), and nonsense variants (16.2%, 324/1995), while for the *MSH2* gene, nonsense variants were the most frequent (31.4%, 608/1935) (*p* > 0.05) (Table 1).

### 3.2. Cumulative Cancer Incidence by Gene and Type of Genetic Variant

**Figure 1:**
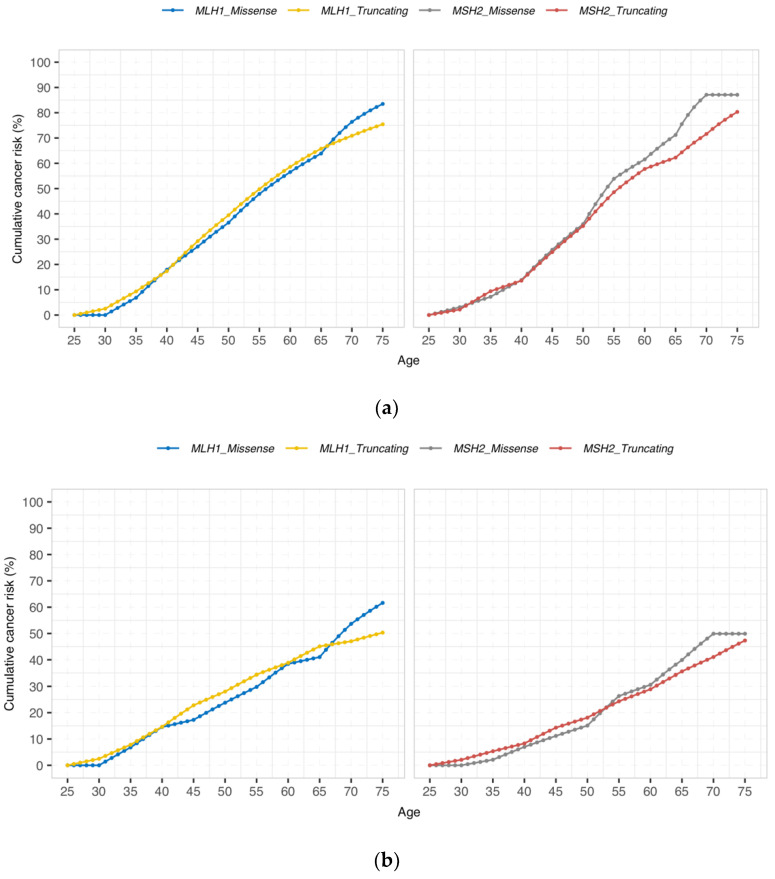

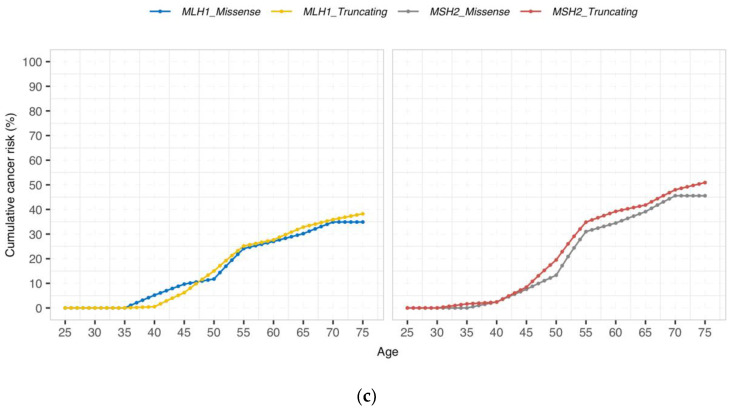
Cumulative incidence of (**a**) any cancer, (**b**) colorectal, and (**c**) endometrial cancer by gene and type of variant. There were no significant differences between carriers with missense/aberrant splicing versus truncating variants at any age in any groups (*p* > 0.05 for all comparisons).

The cumulative incidences by gene for any cancer, CRC, and endometrial cancer are detailed in Table 2 and illustrated in Figure 1. There were no significant differences between carriers with missense/aberrant splicing versus truncating variants at any age in any group. Moreover, no differences which could be considered non-significant trends were observed (*p* > 0.05 for all comparisons).

Cumulative incidences for any cancer at 50 years in *path_MLH1* carriers with truncating or missense/aberrant splicing variants were 39.5% (95% CI (34.5–44.5)) and 36.5% (95% CI (26.6–46.5)), respectively, and in *path_MSH2* carriers, 35.2% (95% CI (29.3–41.1)) and 36.0% (95% CI (23.9–48.0)), respectively. Corresponding cumulative incidences for CRC were 28.0% (95% CI (23.3–32.7)) versus 23.8% (95% CI (14.6–33.0)) for *path_MLH1* carriers and 18.1% (95% CI (13.3–22.9)) versus 15.1% (95% CI (6.4–23.8)) for *path_MSH2* carriers with truncating or missense/aberrant splicing variants, respectively. Corresponding cumulative incidences for endometrial cancer were 15.0% (95% CI (10.1–19.9)) versus 11.8% (95% CI (3.5–20.0)) for *path_MLH1* carriers and 19.5% (95% CI (12.9–26.1)) versus 13.3% (95% CI (2.4–24.2)) for *path_MSH2* carriers with truncating or missense/aberrant splicing variants, respectively.

Cumulative incidences for any cancer at 75 years in *path_MLH1* carriers with truncating or missense/aberrant splicing variants were 75.4% (95% CI (69.1–81.8)) versus 83.5% (95% CI (71.4–95.6)), respectively, and in *path_MSH2* carriers 80.3% (95% CI (73.3–87.4)) versus 87.1% (95% CI (75.6–98.6)), respectively. Corresponding cumulative incidences for CRC were 50.3% (95% CI (43.8–56.8)) versus 61.6% (95% CI (45.9–77.4)) for *path_MLH1* carriers and 47.3% (95% CI (39.6–55.1)) versus 49.9% (95% CI (36.4–63.4)) for *path_MSH2* carriers with truncating or missense/aberrant splicing variants, respectively. Corresponding cumulative incidences for endometrial cancer were 38.2% (95% CI (29.0–47.4)) versus 34.9% (95% CI (19.2–50.6)) for *path_MLH1* carriers and 50.9% (95% CI (39.5–62.3)) versus 45.6% (95% CI (25.6–65.6)) for *path_MSH2* carriers with truncating or missense/aberrant splicing variants, respectively.

## 4. Discussion and Conclusions

In contrast to expectations for the hypothesis we tested, carriers of truncating variants of either *path_MLH1* and *path_MSH2* had similar average cumulative incidences of cancers to carriers of missense or aberrant splicing variants affecting the corresponding gene. On average, carriers of both categories of pathogenic variants had the same high cumulative incidences of any cancer for both genes. The numbers of carriers in each of the groups were large enough to detect any major differences. Our findings will be of clinical interest when interpreting the results of genetic testing, and in planning preventive health care interventions in carriers. As reported previously [1,4], path_MSH2 carriers have higher incidence of other cancers than in the colorectum and endometrium, which is also reflected in the current results for carriers of both truncating and missense *path_MSH2* variants. The cumulative cancer incidences for missense *path_MSH2* carriers in the two highest age groups showed variation that was considered likely to be stochastic, reflecting the limited number of observation years.

We have previously reported that pathogenic variants in each of the MMR genes result in different risks for cancers in organs, including the colorectum, endometrium, ovaries, stomach, small bowel, bile duct, pancreas, and upper urinary tract [1]. Previously, only one study with a very limited number of cases attempted to address the issue of whether the type of pathogenic variant also resulted in different cancer risks but was inconclusive [13].

Some MMR gene variants may be associated with partial but compromised function. The POLYPHEN and SIFT algorithms [26,27] attribute distinct degrees of malfunctioning to different missense variants, and there are examples of aberrant splicing and missense variants in the *BRCA*-genes that are associated with intermediate cancer incidences [22,28,29]. We cannot rule out the possibility that the criteria applied by the InSiGHT database to classify variants lack the sensitivity to identify low-risk variants in *MLH1* and *MSH2*. Indeed, a functional study showed that the *MLH1* variant p.K618T that was classified benign by InSIGHT had an intermediate repair capacity of ~35% to 50% [30]. Carriers of such variants may be at moderately increased risk for cancer, but may not be offered appropriate health care. Less penetrant *path_MMR* variants may also present clinically as the autosomal recessive constitutional mismatch repair deficiency syndrome (CCMRD), but *path_PMS2* and *path_MSH6* variants account for the majority of such cases. *Path_PMS2* variants associated with a milder heterozygous phenotype may be overrepresented, since it was shown that heterozygous relatives of CMMRD patients had a lower cumulative colon cancer risk (8.7%) than reported for *path_PMS2* as a whole by the PLSD and others [1,31]. A difference in age at CRC diagnosis was found for *path_PMS2* carriers when stratifying variants into those that lead to loss of RNA expression compared to those for which expression was preserved [32,33], but a similar relationship was not observed in a CMMRD family cohort. Host immune factors may also be involved in determining cancer incidence in LS. Carriers of *path_MLH1* and *path_MSH2* variants develop thousands of mismatch repair-deficient and potentially precancerous gastrointestinal crypts [34,35]. The frequency at which they progress to infiltrating cancers may be largely determined by the host immune system, rather than the nature of the inherited *path_MMR* variant [34,36]. Genetic modifiers may also contribute to the variation in cancer risk and phenotypic variability in *path_MMR* carriers, leading those with such genetic modifiers to be at increased risk of having further cancers [9].

The strengths of our study include its large sample size and its prospective design, but a potential weakness is selection bias at contributing centres that may have failed to identify some low-penetrance variants. We are also aware that there are many other possible categorizations of *path_MMR* variants that could be investigated for differences in associated cancer incidences using the PLSD data, but we hesitate to do so until we have other plausible hypotheses to test. Similarly, we did not test for differences between class 4 and class 5 variants in relation to cancer incidence, as numbers were not large enough to make this comparison.

The penetrance of the pathogenic MMR variants has no bearing on the classification of their pathogenicity. Having recruited sufficient numbers of carriers into PLSD to reach robust conclusions, we examined the hypothesis that missense or aberrant splicing variants may have lower incidence of cancer than truncating *path_MLH1* and *path_MSH2* variants. In contrast to our hypothesis, we found no difference. The results are of practical interest when presenting preventive health care options to carriers of *path_MLH1* and *path_MSH2* variants.

## Figures and Tables

**Table 1:** Categorization of the *path_MLH1* and *path_MSH2* carriers having inherited variants causing truncating or missense/aberrant splicing clinically actionable variants as defined in the InSiGHT database.

Categorization Group	Variant Type	Gene	Number of Carriers	Sum of the FUP Years	Mean of the FUP Years	95% CI
Missense or aberrant splicing	Aberrant Splicing	*MLH1*	233	1829	7.8	(7.1–8.5)
	Aberrant Splicing	*MSH2*	350	2778	7.9	(7.4–8.4)
	Missense	*MLH1*	345	2715	7.9	(7.4–8.4)
	Missense	*MSH2*	117	883	7.5	(6.7–8.3)
	Total		1045	8205	7.9	(7.6–8.2)
Truncating	Canonical Splicing	*MLH1*	501	4709	9.4	(8.9–9.9)
	Canonical Splicing	*MSH2*	185	1635	8.8	(8.0–9.6)
	Exon Deletion	*MLH1*	688	7643	11.1	(10.6–11.6)
	Exon Deletion	*MSH2*	579	4207	7.3	(6.9–7.7)
	Nonsense	*MLH1*	324	2880	8.9	(8.3–9.5)
	Nonsense	*MSH2*	608	4929	8.1	(7.7–8.5)
	Frameshift	*MLH1*	482	3722	7.7	(7.3–8.1)
	Frameshift	*MSH2*	563	4416	7.8	(7.4–8.2)
	Total		3930	34,141	8.7	(8.5–8.9)
Others	Exon Duplication	*MLH1*	1	1	1	(1.0–1.0)
	Exon Duplication	*MSH2*	16	71	4.4	(2.7–6.1)
	Inframe Indel	*MLH1*	85	790	9.3	(8.3–10.3)
	Inframe Indel	*MSH2*	93	811	8.7	(7.7–9.7)
	Initiation Codon	*MLH1*	8	36	4.5	(1.5–7.5)
	Intronic	*MSH2*	3	25	8.3	(2.1–14.5)
	Undefined	*MLH1*	18	249	13.8	(10.7–16.9)
	Total		224	1983		

FUP, follow-up years.

**Table 2:** Cumulative cancer incidences stratified by age, gene, variant, and organ.

		Cumulative Incidences (95% CI)			
	Age	*MLH1* Missense/Aberrant Splicing	*MLH1* Truncating	*MSH2* Missense/Aberrant Splicing	*MSH2* Truncating
Any cancer	30	0 (0–0)	2.5 (0.3–4.7)	3.1 (0–9.1)	2.2 (0–4.6)
	40	17.9 (9.3–26.5)	17.3 (13.1–21.6)	13.8 (4.1–23.5)	13.6 (8.9–18.2)
	50	36.5 (26.6–46.5)	39.5 (34.5–44.5)	36.0 (23.9–48.0)	35.2 (29.3–41.1)
	60	56.6 (44.6–68.4)	58.6 (53.4–63.9)	61.6 (49.5–73.6)	57.8 (51.4–64.1)
	70	76.4 (63.6–89.2)	71.0 (65.1–76.7)	87.1 (75.6–98.6)	71.6 (64.4–78.8)
	75	83.5 (71.4–95.6)	75.4 (69.1–81.8)	87.1 (75.6–98.6)	80.3 (73.3–87.4)
Colorectal cancer	30	0 (0–0)	2.5 (0.3–4.6)	0 (0–0)	2.1 (0–4.4)
	40	14.5 (6.5–22.6)	14.6 (10.6–18.6)	7.0 (0.3–13.6)	8.3 (4.5–12.1)
	50	23.8 (14.6–33.0)	28.0 (23.3–32.7)	15.1 (6.4–23.8)	18.1 (13.3–22.9)
	60	38.4 (26.5–50.4)	38.9 (33.7–44.0)	30.6 (19.7–41.5)	28.9 (23.3–34.5)
	70	53.7 (39.0–68.3)	47.0 (41.2–52.8)	49.9 (36.4–63.4)	41.1 (34.2–48.0)
	75	61.6 (45.9–77.4)	50.3 (43.8–56.8)	49.9 (36.4–63.4)	47.3 (39.6–55.1)
Endometrial cancer	30	0 (0–0)	0 (0–0)	0 (0–0)	0 (0–0)
	40	5.2 (0–10.9)	0.5 (0–1.5)	2.5 (0–7.2)	2.4 (0–5.0)
	50	11.8 (3.5–20.0)	15.0 (10.1–19.9)	13.3 (2.4–24.2)	19.5 (12.9–26.1)
	60	27.0 (13.9–40.1)	27.7 (21.0–34.3)	34.5 (17.6–51.3)	39.2 (30.3–48.1)
	70	34.9 (19.2–50.6)	35.9 (27.6–44.2)	45.6 (25.6–65.6)	48.0 (37.4–58.5)
	75	34.9 (19.2–50.6)	38.2 (29.0–47.4)	45.6 (25.6–65.6)	50.9 (39.5–62.3)

CI, confidence interval.

## Data Availability

The cancer risk algorithm is available at the PLSD website (www.plsd.eu, accessed on 12 February 2021) that is based upon the results presented in this report and enables interactive calculation of remaining lifetime risks for cancer in any LS patient by giving their age, gender, and gene variant.
